# Fire Blight Control: The Struggle Goes On. A Comparison of Different Fire Blight Control Methods in Switzerland with Respect to Biosafety, Efficacy and Durability

**DOI:** 10.3390/ijerph120911422

**Published:** 2015-09-11

**Authors:** Michele Gusberti, Urs Klemm, Matthias S. Meier, Monika Maurhofer, Isabel Hunger-Glaser

**Affiliations:** 1 Institute of Integrative Biology Zurich, Plant Pathology Group, Swiss Federal Institute of Technology, Zurich CH-8092, Switzerland; E-Mail: michele.gusberti@usys.ethz.ch; 2 Swiss Expert Committee for Biosafety, Bern CH-3003, Switzerland; E-Mail: info@ursklemm.ch; 3 Research Institute of Organic Agriculture (FiBL), Frick CH-5070, Switzerland; E-Mail: matthias.meier@fibl.org

**Keywords:** *Erwinia amylovora*, disease control, pesticides, breeding, genetic engineering, biosafety

## Abstract

Fire blight (FB), caused by *Erwinia amylovora*, is one of the most important pome fruit pathogens worldwide. To control this devastating disease, various chemical and biological treatments are commonly applied in Switzerland, but they fail to keep the infection at an acceptable level in years of heavy disease pressure. The Swiss authorities therefore currently allow the controlled use of the antibiotic streptomycin against FB in years that are predicted to have heavy infection periods, but only one treatment per season is permitted. Another strategy for controlling *Erwinia* is to breed resistant/tolerant apple cultivars. One way of accelerating the breeding process is to obtain resistant cultivars by inserting one or several major resistance genes, using genetic engineering. To date, no study summarizing the impact of different FB control measures on the environment and on human health has been performed. This study consequently aims to compare different disease-control measures (biological control, chemical control, control by antibiotics and by resistant/tolerant apple cultivars obtained through conventional or molecular breeding) applied against *E. amylovora*, considering different protection goals (protection of human health, environment, agricultural diversity and economic interest), with special emphasis on biosafety aspects. Information on each FB control measure in relation to the specified protection goal was assessed by literature searches and by interviews with experts. Based on our results it can be concluded that the FB control measures currently applied in Switzerland are safe for consumers, workers and the environment. However, there are several gaps in our knowledge of the human health and environmental impacts analyzed: data are missing (1) on long term studies on the efficacy of most of the analyzed FB control measures; (2) on the safety of operators handling streptomycin; (3) on residue analyses of *Equisetum* plant extract, the copper and aluminum compounds used in apple production; and (4) on the effect of biological and chemical control measures on non-target fauna and flora. These gaps urgently need to be addressed in the near future.

## 1. Introduction

Fire blight (FB), caused by *Erwinia amylovora*, is most probably the most destructive bacterial disease in apple and pear orchards worldwide. The bacterium infects plants of the Rosaceae family, such as species belonging to the genera *Malus*, *Pyrus*, *Cydonia*, *Crataegus*, *Sorbus*, *Cotoneaster*, *Amelanchier*, *Eriobotrya*, *Heteromeles*, *Mespilus*, *Photinia*, *Rhaphiolepsis*, *Stranvaesia* and *Pyracantha* [1]. Other rosaceous plants are reported to be potential hosts for *E. amylovora* “*under unusual circumstances*”, such as some species of the genera *Aruncus*, *Fragaria*, *Rosa*, *Rubus*, *Spirea and Prunus* [1,2]. Some of these species are included in the Red List for Swiss fern and flora, that contains endangered species in Switzerland [3]. *Cotoneaster* species, especially those belonging to *Cotoneaster salicifolius* group (*C. salicifolius*, *C. floccus*, *C. waterei*, *C. bullatus*, *C. franchetti*) are late blooming plants and so they are very important to the epidemiology of *E. amylovora* [4]. The eradication of about 90% of apparently healthy *Cotoneaster* is expected to be essential to maintain the disease pressure at as low a level as possible [4].

*Erwinia amylovora*, a gram-negative enterobacterium, overwinters in living tissues at the margins of cankers and becomes active in spring when suitable climatic conditions are reached: a threshold temperature of 18.3 °C has been suggested [5]. The growth of *E. amylovora* in axenic liquid media is inhibited at pH = 5, and at a lower pH bacterial growth is nearly abolished [6]. The bacterium is transmitted to healthy tissues mainly by insects, wind and rain splashes. The optimum climatic conditions for the multiplication of the bacterium are between 23 and 30 °C (reviewed in [7]). The bacterium infects the flowers of the host using the type III secretion system primarily during the blooming period of the trees [8,9]. Characteristic symptoms are the typical “shepherd’s crook” of the twigs and the presence of milky-yellowish bacterial exudate on the infected tissues.

*Malus × domestica*, the domesticated apple plant, originates from central Asia, probably from a spontaneous cross between *M. sieversii* and the European apple species *M. sylvestris* [10,11,12,13]. The selection of the first apple cultivars in monasteries and castles was described in the Middle Ages [14,15]. Successive cross-breeding between apple cultivars led to the generation of modern apple cultivars [14]. Production of new apple cultivars enabled also a change in the agricultural practices used in the orchard. Standard trees, planted as single trees (of different cultivars) and surrounded by a savannah-like environment (low density) were replaced by dwarf trees planted in orchards organized in rows (high density orchards) [16,17]. This may have an impact (1) on ecological aspects: standard tree orchards show a greater biodiversity of birds, arthropods and plants compared to high density orchards compared to dwarf tree orchards (both integrated and organic production) [16,17]; (2) on plant care: lower plant canopies facilitate the work in the orchard (pruning, disease control measure and harvest); and (3) on the procession of the product apple: dwarf trees are used in both integrated and organic apple production for dessert apples, while standard trees are generally used in integrated apple production and the fruit is mostly used for juice and cider production [18].

In Switzerland, about 141,247 tonnes of apples over a surface area of about 4000 ha dwarf tree orchards are produced annually [19]. About 90% of Swiss apple production follows the IP-Suisse guidelines and about 5% follows the BIO-Suisse guidelines. Forty percent of apple production is generated by “Golden Delicious” and “Gala”, followed by “Braeburn” (9.4%), the Jonagold group (6.3%), “Maigold” (4.9%), “Gravenstein” (3.3%), “Idared” (3.1%), “Topaz” (2.6%) and “Milwa” (2.4%) as the next most frequently planted apple cultivars (for a more complete list see [20]). Thus, most of the apple production is generated using FB susceptible apple cultivars [21].

The first observation of *E. amylovora* in Switzerland dates from 1989 on *Cotoneaster* sp. and the first outbreak in apple and pear orchards occurred in 1991 (reviewed in [7,22]). Due to the highly destructive nature of this pathogen, quarantine and eradication measured were promptly adopted [22]. After these first observations, the disease spread through most of the northern and central Swiss regions, reaching a peak in 2007 [7]. As a consequence, about 100 hectares of dwarf pome orchards and about 40,000 standard trees were eradicated at a cost of about CHF 50 million [23]. In 2008 the Federal Office for Agriculture (FOAG) permitted the use of streptomycin in pear and apple orchards to control FB under strict rules. In addition to streptomycin, other control measures (biocontrol agents and chemical products) are also being used against FB depending on the agricultural production system (organic or integrated production) adopted in the orchard.

Another important control measure is the breeding of resistant or tolerant apple cultivars. However, not many resistant cultivars have yet been produced. In the last two decades, investigation has begun into the control of FB through wild and crab apple resistance genes, such as genes found in *Malus × robusta* 5, *M. × domestica* “Evereste”, *M. fusca* and *M. baccata* [24,25]*.* Studies have also been performed to find quantitative trait loci (QTLs from *M. × domestica* “Enterprise”, “Rewena”, “Fiesta”, “Florina” and *M. floribunda* 821) for FB resistance in cultivars showing low disease severity [26]. Fire blight-resistant apples can be created through classical breeding or genetic engineering methodology. Classical breeding involves two different parent plants that are crossed to generate thousands of seedlings. These are screened visually and molecularly for the traits of interest. However, despite the use of molecular tests, classical breeding is still considered a time-consuming and laborious process [27,28]. For example, the Swiss apple breeding program, as performed by Agroscope in Wädenswil [27], consists of different selection steps starting from about 25 cross combination every year. Plants are then screened (visually and molecularly) for plant growth characteristics and for scab (*Venturia inaequalis*), powdery mildew (*Podosphaera leucotricha*) and FB resistance/tolerance traits. *Malus x domestica* “Ladina” (“ACW 14959”; *FB_F7* QTL) was created in 1999 at Agroscope in Wädenswil from a cross between “Topaz” and “Fuji”, which shows *Rvi6* scab resistance, low mildew susceptibility and FB tolerance. This cultivar took 14 years to reach the market testing stage [29].

A second strategy to produce FB-resistant apple plants is to use approaches based on genetic engineering, such as the cisgenesis approach [30,31,32]. Cisgenesis is the insertion of a gene (composed of its native promoter, coding and terminator sequences) from a sexually compatible and crossable species [33]. Briefly, the generation of cisgenic plants resistant to FB comprises the identification and isolation of a resistance gene (which may take 3–5 years) followed by the construction of a plasmid vector [30,31,34]. Then, under *in vitro* conditions, leaf cells of an apple cultivar (e.g., “Gala”) are transformed with an *Agrobacterium* strain carrying the plasmid vector constructs. After these steps, the plants (transgenic at this stage) are subjected to recombination activity to cleave the marker-genes and to subsequent subculturing in different media to select marker-free plants [30,31,34]. An example of this approach is a “Gala” in which the *FB_MR5* FB-resistance gene, from the crab apple *M. × robusta* 5, has successfully been inserted [32] and more recently the first cisgenic lines have been created [35,36].

In Switzerland, the handling of GMOs is regulated by the Gene Technology Act [37]. This law aims to protect human/animal health and the environment. The Gene Technology Act defines GMOs in Article 5 paragraph 2 as “*organisms in which the genetic material has been altered in a way that does not occur under natural conditions by crossing or natural recombination*”. The Release Ordinance lists methods involved in the genetic transformation of organisms (Art. 3 letter d, Annex I), for example nucleic acid recombination and certain cell fusion or hybridization techniques [38]. According to the current Swiss regulations, cisgenic plants are regarded as GMOs.

In general, for proper fruit orchard management, the application of pesticides (chemical or biological) and the integration of tolerant cultivars are both used in parallel. However, to date, no study summarizing the impact of FB control measures on the environment, on human and on animal health has been performed. This report therefore aims to compare different FB control measures (biological control, chemical control, control by antibiotics and by resistant cultivars obtained through classical breeding or genetic engineering) in the most common agricultural practices (organic and integrated production) in Swiss apple orchards, looking at the different protection goals but with a special emphasis on biosafety aspects.

## 2. Methods

### 2.1. Identification of Protection Goals

The use of pesticides in agriculture in Switzerland is regulated by the Plant Protection Products Ordinance (PlantPPO) [39]. Pesticides (Art. 4, paragraph 5) should show an acceptable efficacy, have no effect (short or long term) on human and animal health, should not show any unacceptable effect on the product (e.g., fruit or vegetables), and should not impose unacceptable effects on the environment. At the environmental level, particular importance has been attached to the fate and distribution of the pesticides in water, soil and air, as well as their impact on non-targeted species, biodiversity and the ecosystem. Moreover, residues of pesticides should not have any harmful effect on human and animal health or on the environment [39].

To comply with all these rules, different protection goals (PG) have been defined for FB control in apple orchards. The main goal, PG1, defined as maintaining FB-free orchards and environment, has been investigated for feasibility, durability and efficacy of the FB control measure considered. The second, PG2, has been defined as protection of human health. For this PG, the effects of the FB control measure on operators and on consumers have been investigated. The third, PG3, concerns the protection of the environment. For this, information on the effect of FB control measures on exposed animals, on soil and water environments, and on biodiversity have been collected. Unacceptable effects on the product have been investigated for PG4, defined as protection of economic interests. To this end, factors such as consumer acceptance and marketability of the product “apple” were considered. The preservation of agricultural diversity was summarized under PG5. The impact of the FB control measures has therefore been investigated with respect to apple cultivar diversity and to the diversity of the agricultural practices (organic and integrated apple production; standard and dwarf apple trees) applied in the orchard.

### 2.2. Identification of the Most Representative FB Control Measures Applied in Switzerland

#### 2.2.1. Preventive FB Control Measures

The following preventive FB control measures are commonly used in Switzerland: (1) management of wild and ornamental host-plants (e.g., eradication of *Cotoneaster salicifolius* group) [4]; (2) winter pruning of cankers, flowers or fruit mummies [40]; (3) elimination of highly infected plants [40]; (4) hail protection netting; (5) prohibition of nomadic beekeepers during the flowering period of apple and pear plants [41]; (6) use of tolerant apple cultivars [42]; and (7) use of tolerant or resistant apple rootstocks (e.g., CG rootstock series [43]).

#### 2.2.2. FB Control Measures Applied in Organic and Integrated Swiss Apple Production

In order to control this bacterial disease, different chemical and biological compounds are routinely used in Switzerland, depending on the agricultural practices applied in the orchard (integrated or organic apple production). The most commonly used pesticides include *Aureobasidium pullulans*, a yeast-like fungus limiting the growth of *E. amylovora* in pome fruit flowers, which is recommended (Switzerland) to be applied during flowering, the day before infection conditions are predicted, according to disease forecasting models with a maximum application of 12 kg·ha^−1^·year^−1^ [44,45]. Copper compounds are known to inhibit the germination of fungi and oomycetes as well as the growth of bacteria [46,47]. Application of copper compounds at the bud break stage of apple (BBCH 51–53, max. 4 kg metallic Cu·ha^−1^·year^−1^) is recommended if infection events have occurred in previous years [48,49]. Products containing aluminum sulfate and *Equisetum* extract are applied from the balloon stage (BBCH 59) to the end of flowering (BBCH 67) at a concentration of 5% (max. 8 kg·ha^−1^·year^−1^) [45]. While *Equisetum* essential oil is known to have antimicrobial effects [50], the mechanism of action of aluminum is not entirely known. Suggested modes of action include medium acidification, inhibition of ion transport and ATP synthesis (reviewed in [51,52]). Potassium aluminum sulfate compounds are known to be astringent and aseptic substances that inhibit the growth of bacteria, fungi and oomycetes [53,54]. Potassium aluminum sulfate was first used to decrease human waterborne disease infections by flocculation and water acidification processes (reviewed in [55]). Three applications of this product (max. 20 kg·ha^−1^·year^−1^) are allowed in Switzerland against *E. amylovora*; a fourth can be performed after a hail event [56,57]. Since the discovery of streptomycin in the early 1940s [58], this antibiotic has been used in medical, veterinary and agricultural settings. In Switzerland the strict and controlled use of streptomycin sulfate-containing products to combat *E. amylovora* received exceptional authorization in 2008 [57,59]. Between 2008 and 2009 three streptomycin applications year^−1^ were authorized, while between 2010 and 2013 this was reduced to two streptomycin applications year^−1^ [57,60]. However, since 2014 only one treatment year^−1^ has been permitted [59].

Besides the above mentioned products and compounds the following products are also applied in Switzerland: products based on the biocontrol bacterium *Bacillus subtilis* as well as the plant defense activators Acibenzolar-S-Methyl and Laminarin and the plant growth regulator Prohexadione-Ca. These products were not considered in this study.

In addition to biological and chemical products, FB-resistant/tolerant apple cultivars may further decrease the disease pressure in the apple orchards. Fire blight-resistant/tolerant apple plants may be created using classical breeding or genetic engineering methodology [27,29,31,32,34]. The single gene tolerant (assumption not confirmed) classically bred apple “Ladina” has recently been released onto the market [29], while a transformed “Gala”, containing the *M. × robusta 5* resistance gene *(FB_MR5*), has recently been published [32].

### 2.3. Data Collection

Information and empirical data on each FB control measure in terms of the specified PG were obtained through literature searches and from interviews with experts.

Literature searches were performed as follows: (1) manual searches (e.g., Google, Google Scholar and Web of Science) were used to find all potentially relevant and internationally peer-reviewed articles dealing with *E. amylovora*, FB control, antibiotics in agriculture, environmental impact of chemicals, classical breeding, and cisgenesis; (2) Ph.D. and Master’s theses dealing with *E. amylovora* or FB control were also included; (3) further information on specific products/methods was obtained from the European Food Safety Authority (EFSA) and from the relevant Swiss centers (*i.e.*, Federal Office for Agriculture: FOAG; Federal Office for the Environment: FOEN; Federal Food Safety and Veterinary Office: FSVO; Swiss State Secretariat for Economic Affairs: SECO; Agroscope; Research Institute of Organic Agriculture: FiBL). Additional information was collected by interviewing experts (*n* = 16) who could contribute to the study according to their field of expertise by providing more knowledge of how each FB control strategy could support or interfere with each PG. Interviews were performed with experts on ecotoxicological risks (soil micro- and macroorganisms), microbiological and biotechnological risks (streptomycin and genetically engineered organisms), pesticide authorizations and application (legislative procedures and praxis), toxicological risks for animals and humans (pesticides, streptomycin and antibiotic resistance), breeding (classical breeding and genetic engineering), agricultural practices (organic and integrated production; standard and dwarf trees), and forest ecology. In addition to being interviewed, experts were asked to complete a questionnaire to assess the magnitude and probability of damage caused by the selected FB control measure to the identified PG in a worst-case scenario. The magnitude of damage to each PG was assessed using a four-step scale (1. very low/negligible; 2. low; 3. high; 4. very high). Similarly, a four-step scale was also used to assess the probability of damage occurrence (1. highly unlikely; 2. unlikely; 3. likely; and 4. most likely; for examples see Table 1 and Table 2).

**Table 1:** Example of the assessment of durability of the fire blight (FB) control measure investigated.

Durability of FB Control Measure																
Effect	Negligible				Low				High				Very High			
Development of resistance in *Erwinia* following intensive use of the product	No resistance or tolerance development				Less than 10% of the strains would develop resistance or tolerance to the product after more than 20 years				More than 50% of the strains become resistant in 10–20 years				All FB strains become resistant in the next 5 to 10 years			
Probability of damage occurrence ^a^	1	2	3	4	1	2	3	4	1	2	3	4	1	2	3	4

**^a^**: Probability scale used: (1) damage possible, but under comparable conditions has never occurred (=highly unlikely); (2) damage possible in individual cases under particularly unfavorable conditions; (3) damage under comparable conditions already known (occurrence in 10%–50% of the cases); (4) occurrence of damage frequent (>50% certainty).

**Table 2:** Example of the assessment of the impact of the fire blight (FB) control measure on soil organisms.

Impact of FB Control Measure on Soil Organisms																		
Effect	Concerned?		Negligible				Low				High				Very High			
Soil organisms	Y	N	Population of some exposed species temporarily lowered. Regeneration possible within days.				10% of the exposed species temporarily reduced. Regeneration possible within weeks.				20%–50% of the exposed species die. Recolonization possible within one year.				>50% of the exposed species die. Repopulation possible only after extensive soil remediation.			
	
	
Probability of damage occurrence ^a^			1	2	3	4	1	2	3	4	1	2	3	4	1	2	3	4

**^a^**: Probability scale used: (1) occurrence of damage inconceivable (=highly unlikely); (2) occurrence of damage possible but unlikely (in exceptional cases possible); (3) occurrence of damage quite possible; (4) occurrence of damage most likely (>90% certainty).

## 3. Results and Discussion

### 3.1. Impact of Fire Blight Control Measures on the Chosen Protection Goals

#### 3.1.1. PG 1: Fire Blight-Free Orchards and Environment

For the PG of FB-free orchards and environment the information on biosafety aspects extracted from the literature was in agreement with that obtained from the experts. All chemical and biocontrol products included in the study, as well as the first classically bred tolerant cultivars, are already on the market.

The durability of all chemical and biological FB control measures, because of the stringent Swiss application rules (e.g., max. 1 application of streptomycin containing products year^−1^), is considered to be high. However, more permissive legislation on the application of streptomycin may lead to selection of streptomycin-resistant genes in the bacterial community. In *Erwinia*, the identification of streptomycin-resistant strains has already been observed, for example in the USA [61,62], Egypt [63], New Zealand [64], Israel [65], Canada [66], Lebanon (reviewed in [67]), Syria [68] and Mexico [69].

The experts indicated some uncertainty about the durability of single gene-based FB resistance in classical as well as cisgenic cultivars. The main problem observed with the *FB_F7* QTL conferring FB-tolerance to the classically bred apple “Ladina” is its varying tolerance level [29]. Experts stated that, on average, plants containing the *FB_F7* QTL are more tolerant than plants without it, although some genotypes may still be rather FB-susceptible even with the QTL. Thus, the presence of the markers flanking this QTL is not a guarantee of the resistance/tolerance of the plant, and shoot inoculation experiments with FB must still be performed. However, testing “Ladina” under outdoor conditions confirmed FB tolerance and this variety is already undergoing first market tests. The first genetically engineered (cisgenic) FB-resistant apple is a “Gala” containing the *M. × robusta* 5 resistance gene. However, some *Erwinia* strains which are virulent to *M. × robusta* 5 have already been observed in North America (*i.e.*, Ea266 and Ea273) [70]. Moreover, a naturally-occurring single nucleotide polymorfisme (SNP) in *avrRpt2* gene of *Erwinia* is enough to overcome the FB resistance provided by *M. × robusta* 5 [71,72]. Experts were aware of these potential problem and researches on different FB-resistance genes to decrease the risk of *FB_MR5* breakdown are underway. The use of plants bearing multiple resistance genes (pyramid) may prolong the plant’s durability, as suggested for different plant-pathogen systems (reviewed in [73]). However, apple cultivars with multiple FB-resistance genes do not yet exist.

The efficacy of the FB control measures varies greatly depending on the product/method considered. The biological control agent *A. pullulans* showed the best results performing a detached flower infection assay (88%–93% FB control), whereas 89% reduction was obtained with streptomycin sulfate [44]. However, long-term field studies using *A. pullulans* showed an average FB reduction between 73% and 78% [6,74]. Copper compounds showed the highest variability among the analyzed products [6,75]. Copper sulfate showed an efficacy ranging from 34% to about 60% and copper oxychloride showed an efficacy between 47% and 58% [6,76]. The highest efficacy among the analyzed copper compounds was observed with copper hydroxide (75%–88%) [6,21]. Field experiments using aluminum sulfate showed that the average efficacy varied between 51% and 65% [6,74]. Potassium aluminum sulfate was tested two consecutive years in Germany (2010–2011) and the efficacy has been found to be highly variable (56%–85%) between the tested years [77]. However, an average efficacy of potassium aluminum sulfate of 73% was shown in 8 field trials [74]. Experiments carried out in Germany, Switzerland and in Austria could show that the efficacy of potassium aluminum sulfate is about 10%–15% lower than the efficacy of streptomycin [78]. The problem evidenced by the experts with the aluminum compounds is that the efficacy decreases quite fast after the application and it is washed away even after slight rainfall. Application of streptomycin reduced the flower infections in average by 76%–89% [21,74,76,79,80,81]. Nevertheless, efficacies between 60% and 97% have been observed [82,83].

Results of 4 years of greenhouse and field tests with the single-gene based FB-tolerant classically bred apple “Ladina” [29], showed about a 30% field flower infection score with *E. amylovora* (“Gala Galaxy” around 70%), a score of about 10% after artificial greenhouse flower infection (“Gala Galaxy” about 60%) and between 10% and 15% lesion length after greenhouse shoot infections (“Gala Galaxy” about 85%), corresponding to 60% and 75% efficacy for flowers and shoot infections, respectively. Experts suggest that the use of FB-tolerant cultivars (e.g., “Ladina”) enables the reduction of the pathogen pressure to an acceptable level. Moreover, “Ladina” can show symptoms under high infection pressure in an orchard, but infected tissue can easily be removed, reducing therefore the pathogen pressure. However, epidemiologically speaking these trees hiding bacteria also in healthy looking tissue may contribute to the infection of other orchards or other standard trees. Thus, some risks of spreading of the pathogen may still exist.

*Malus × domestica* “Gala” lines transformed with the *FB_MR5* gene inoculated with two *E. amylovora* strains harboring the wild type *avrRpt2* gene (Ea1189 and Ea222_JKI [84,85]) displayed on average between 0% and 4% stem necrosis, while the control plants (“Gala”) showed between 58% and 68% stem necrosis [32], corresponding to an efficacy between 93% and 100%.

For this PG, data gaps were associated with the fact that two-gene FB-resistant cultivars do not yet exist either for classically bred or cisgenic apples, so there is no reliable information on the durability or efficacy of resistance based on two genes. Moreover, long term field studies using most of the analyzed FB-control measures are scarce or missing. Future research on this topic is therefore necessary to better assess the efficacy of these products/methods in the frame of apple production.

#### 3.1.2. PG 2: Human Health

The influence of FB control measures on human health has been investigated for operators and consumers. No problems are expected for either group when using classically bred apple cultivars, chemical or biological FB control measures.

No risks for operators handling *A. pullulans* have been predicted as long as it is used according to the manufacturer’s instructions. No Acute Reference Dose (ARfD) or Acceptable Operator Exposure Level (AOEL) have been defined for this product [86]. A potential increased risk of asthma and allergy upon repeated contact has been suggested [45,87], but no allergic reactions or sensitization have been observed in operators handling the product [86]. Moreover, experts confirmed that the R- and S-phrases written on the package are mainly for the citric acid component, which is why eye protection is recommended. Since *A. pullulans* might induce an allergic reaction, exposure must be minimized (e.g., using respiratory, eye and skin protection). The AOEL of copper has been set at 0.072 mg Cu·kg^−1^·body weight day^−1^ [88]. Without protection measures, dermal adsorption has been estimated at about 10% and oral adsorption has been assumed to be 100% [88]. If gloves and coveralls are used during mixing, loading and the application of the product, about 60% (tribasic copper sulfate) of the AOEL can be reached (estimated by model German 8 ha; 2 kg Cu·ha^−1^) [88]. Improper use may cause skin irritation, and allergic reactions may also be expected after prolonged skin contact [89]. No ARfD has been defined for aluminum sulfate or *Equisetum* extract-containing products [90]. Instead of an AOEL, a reference value of 0.002 mg·kg^−1^·body weight day^−1^, based on the Tolerable Weekly Intake (TWI) of 1·mg·kg^−1^·body weight day^−1^ corrected for the oral absorption rate (1%), has been established [90]. Considering the use of the substance, the calculated exposure without personal protective equipment was 19%–23% of the reference value for strongly mechanised applications. If gloves were used, an exposure of about 2% of the reference values was defined [90]. Exposure to aluminum (at low doses and over a long period) have been connected to Alzheimer’s disease (reviewed in [91]) and other potential side effects [92]. Accumulation of aluminum in the brain, bones and kidney (animal oral exposure) has been documented [90]. However, experts consider the connection between the aluminum compounds and Alzheimer’s disease in the context of plant protection to be tenuous. The handling of potassium aluminum sulfate is not regarded as harmful even though it may cause lung, skin and eye irritation [93]. The AOEL of streptomycin has been set at 0.01 mg streptomycin·kg^−1^·body weight day^−1^ and the ARfD at 0.05 mg streptomycin·kg^−1^·body weight day^−1^ [94]. The use of streptomycin in human medicine in Switzerland is restricted to treating patients with multidrug-resistant tuberculosis (MDR *Mycobacterium tuberculosis*) [95]. In humans, streptomycin shows a low degree of skin absorption (~1%), it is not a skin or eye irritant, it does not accumulate, and it is rapidly excreted from the body [94]; thus, effects on the operators are not expected if the antibiotic is used in conformity with the manufacturer’s instructions (use of gloves, eye protection, respiratory protection and protective clothing). However, if protection measures are neglected and operators are exposed, the selection of resistant bacteria in the worker would be expected, and these may be transmitted to other people. So far, no analyses of streptomycin or multi-antibiotic resistance in the bacterial flora of operators or their families have been performed; this clearly should be investigated in the future. No negative effects on operators are predicted when using classically bred FB-resistant/tolerant apple cultivars such as “Ladina”. For the genetically engineered cisgenic “Gala”, experts judged the risk to operators to be negligible as scientific biochemical and genetic data show that this breed can be taken for employment safety assessment equivalent to other breeds obtained by classical selection.

For consumers, no risks were predicted by using of *A. pullulans* [86], copper [88], aluminum sulfate [90], or potassium aluminum sulfate [56]. Nevertheless, residues of copper sulfate and aluminum sulfate compounds have been assessed for grape production and for greenhouse cut flower production, respectively. *Equisetum arvense* plant extract was evaluated by the [96] and some potential impacts of this substance on human and animal health have been suggested (e.g., digestive problems due to silicates, possible accumulation of alkaloids resulting in premature childbirth, nervous disorders, headaches, loss of appetite, or difficulties in swallowing). Moreover, chronic ingestion of *Equisetum* may lead to decreased thiamine levels in humans, which however is reversible [50]. The possible impacts on human health identified would be of relevance only if *Equisetum* extract residues were present on apples at harvest; however, there is a lack of studies in this area. The Acceptable Daily Intake (ADI) for streptomycin has been set at 0.01 mg·kg^−1^·body weight day^−1^ [94]. In 2008, antibiotic residues in Swiss apples were measured/quantified at the end of the season [97]. These residues ranged between 0.0005 and 0.009 mg·kg^−1^, thus no risks to consumers are expected. The use of antibiotics during the blooming period of apple and pear trees is connected to the contamination of honey [98]. Bees can fly for many kilometers (up to 13 km in a US study) to find their food source [99], although an average radius of about 3 km has been suggested [100]. Thus, contaminated honey may also be found in regions where no antibiotics were applied [98]. In Switzerland in 2011, honey with a streptomycin content higher than the tolerance value was found and about 9400 kg of honey was destroyed [101].

Because of the limited availability of disease resistance genes in currently used apple cultivars, the development of FB-resistant/tolerant apple plants largely relies on FB resistance genes found among wild and crab apple species. This implies that “wild” traits will be incorporated in the breeding process (reviewed in [102]). Allergens and toxic compounds may therefore also be accidentally incorporated into the newly bred cultivars, potentially affecting consumers. However, current regulations do not require any tests for allergens or toxic compounds before a “new” apple cultivar can be released onto the market [103]. Moreover, not every apple has the same allergenic potential: “Santana”, for example, is very low in allergen while “Golden Delicious” is highly allergenic.

No investigations have so far been made of the consumer safety of the cisgenic FB-resistant “Gala”. In the case of the *Rvi6* scab-resistant cisgenic “Gala”, experts suggest that the risk is probably low since consumers are already eating fruit from cultivars carrying the *Rvi6* resistance gene such as “Florina” and “Topaz”. However, the FB-resistant “Gala” contains a resistance gene originating from a wild apple. If, after the end of the moratorium, a cisgenic FB-resistant apple cultivar were launched on the market, the studies of consumer (and environmental) safety required would depend heavily on how cisgenic plants will be regulated in the future. A major problem is that it is not yet clear whether cisgenic plants will be classified as GM or not in the future. If cisgenesis is considered equivalent to classical breeding, the risks to consumers will also be regarded from the regulatory point of view as similar and no further studies of consumer safety will be necessary. However, if cisgenic plants are considered to be GMO, numerous risk studies, including compositional analysis [60], toxicological safety and nutritional assessments [62], post-market monitoring [63], and environmental risk assessment [64], will have to be performed before a cisgenic FB-resistant apple cultivar would be authorized.

Gaps: Data on the copper residues reported by the EFSA [88,104] refer to grape and tomato cultivations and those for aluminum to greenhouse cut flower production [90]; residue analyses for these products as part of apple production must therefore be performed. Moreover, information on the safety of operators using streptomycin is clearly missing. There are no studies available on antibiotic resistance in the bacterial flora of operators, nor is it known how strictly the safety protocols are really followed.

#### 3.1.3. PG 3: Protection of the Environment

The impact of FB control measures on the environment in terms of exposed animals, soil, water and biodiversity has also been investigated.

For exposed animals, no risks are expected when using classically bred and cisgenic apple cultivars [105,106,107]. While only a low risk for some exposed animals has been suggested for *A. pullulans* [86], applications of copper and aluminum compounds over a long period may pose risks to mammals, birds, aquatic and soil organisms, and bees [90,96,104,108,109]. However, regarding copper, the information delivered to EFSA were not sufficient to assess its long term effect to these organisms [104]. The most susceptible farm animal to copper is the sheep (acute toxicity: 20–100 mg Cu·kg^−1^ body weight), but also some dog’s breed (e.g., Bedlington Terriers) are susceptible to this metal and chronic intoxication are more likely to occur with low intake of molybdenum and sulfur [108]. Furthermore, *Equisetum* extract-containing products may increase the risk of thiamine deficiency in animals, which is rarely reversible [50]. The use of streptomycin sulfate has been estimated as posing only a low risk (*i.e.*, the selection of bacteria carrying antibiotic resistance(s)) to exposed animals when used in a restricted manner. Nevertheless, an experiment [110] simulating the drift of streptomycin to nearby pastures after application in apple orchards reported that when sheep were grazing on a pasture contaminated with streptomycin, the percentage of streptomycin-resistant *Escherichia coli* and *Staphylococcus* isolated from anal and nasal swabs was higher than from a control group grazing on uncontaminated grass. The level of resistant strains decreased again to normal level when the animals were no longer exposed to further pressure. Under normal conditions, such close contact of animals with the antibiotic is not allowed [59].

Several investigations of the impact of GM plants on the environment have been performed over recent decades [reviewed in 107]. For example, a National Research Programme (NRP59) study of GM wheat lines expressing different alleles of specific wheat resistance gene *pm3* against *Blumeria graminis* f. sp. *tritici*, or Barley chitinase and glucanase genes involved in general pathogen resistance, showed no significant differences in the arthropod populations associated with GM compared to non-GM wheat cultivars [105,111]. Another comparative study of volatile compounds showed no change in leafminer (*Phyllonorycter blancardella*) attraction to a GM apple *versus* its representative classically bred genotype [106]. An example of an existing cisgenic disease-resistant crop plant that would be of interest to Swiss farmers is the cisgenic potato A15-031, resistant to *Phytophthora infestans*. This line (*Solanum tuberosum* “Desiree” transformed with the *S. venturii Rpi_vnt1.1* resistance gene of the NB-LRR resistance class) was developed in the Netherlands as part the “DurPh” research programme of Wageningen University [112,113]. Due to the specificity of the *Rpi* resistance gene, researchers do not expect any effect on non-target fauna [113]. Experts further stated that negative effects may not arise from the genetic engineering itself, but from the *in vitro* process: the transgenic and subsequent cisgenic lines derive from a single transformed cell.

Risks concerning soil and water were anticipated for copper compounds (summarized in EFSA study [104]) and for aluminum compounds [114], the former due to soil accumulation and the latter due to medium acidification. There is not yet enough knowledge available on the effects of these compounds on soil and water organisms thus more studies are needed. These gaps in knowledge also need to be further analyzed.

At the biodiversity level, no risks were identified for the use of *A. pullulans* [16], streptomycin sulfate [115,116,117,118], and classically bred apple cultivars [119]. However, potential risks to other plants after the application of copper [88], or to plants and aquatic animals for aluminum compounds [114,120,121], have been documented in the literature and were also reported by the experts. It has been noted that if streptomycin is used in a restricted manner, the selection of streptomycin-resistant genes in the environment is possible for a limited period only [115,116,117,118]. However, the uncontrolled use of antibiotic will probably increase the proportion of streptomycin resistance genes in the bacterial population, as observed elsewhere (e.g. [61,62]).

Moreover, it has been suggested that GM plants carry potential risks to biodiversity [122], for example through the consequences of dissemination and naturalization of the inserted gene and the selection of virulent pathogens. Experts agree that the probability of outcrossing between cultivated and wild apple plants should be the same for GM and classically bred FB-resistant apple cultivars. However, no reports on the impact of outcrossing of resistance genes in apple are available.

Data gaps were identified for the effect of *A. pullulans*, *E. arvense* plant extract, copper and aluminum compounds on some exposed animals and plants [86,96,104]. Moreover, there are no residue analyses for copper and aluminum compounds and for the *Equisetum* extract in apple orchards; these should be performed.

#### 3.1.4. PG 4: Economic Interest

Economic interest has been examined in terms of consumer acceptance and marketability of the product.

Consumer acceptance is high for biological control agents (*A. pullulans*), copper and aluminum compounds, and classically bred apple cultivars. For these cultivars, however, breeders must focus on marketing trends: if an apple cross is produced in 2013 it will not reach the market until approximately 2030 or even later and it is not predictable what product preferences consumers will have 15–20 years from now. Other problems of new cultivars also need to be considered (such as superficial skin scald susceptibility after storage in “Ladina”) before a cultivar is fully accepted by the growers, and consequently by consumers. Consumer acceptance of streptomycin sulfate and GM plants is low [123]; however, most consumers do not seem to be aware that antibiotics can be used in integrated apple production and that copper and some other pesticides are applied even in organic apple production.

The potential for marketability of classically bred apples and apples treated with aluminum compounds or streptomycin sulfate is high. However, copper compounds and *A. pullulans* may induce fruit russeting [124,125,126], thereby reducing the marketability of the fruit. All GM plant products entering the market have to be labelled (Art. 10) as “*genetically modified*”. Currently there is a moratorium in place, and thus no GM products are on the market. The adventitious presence of up to 0.9% GM plant material in the product, if used as food or feed, does not have to be indicated. The threshold for unintentional traces of authorized genetically modified organisms in mixtures, articles and products handled directly in the environment is set at 0.1% [38]. Today, some GM products are now authorized for use in animal feed in Switzerland [127], although no GM phytosanitary products, seeds or fertilizers have yet been authorized [128] due to the moratorium that will last until 2017 [129]. In 2005, the Swiss population was asked to vote on the possibility of allowing GMO food to be produced in Switzerland. The population decided to ban the production of GMO food for a period of 5 years, which has since been extended until 2017 [129]. The low acceptance of GMOs and the moratorium in Switzerland on the cultivation of GM plants hampers the further development of cisgenic apples [129].

#### 3.1.5. PG 5: Agricultural Diversity

The impact of FB control measures on the diversity of apple cultivars or agricultural practices, summarized as agricultural diversity, was also assessed.

The efficacy of *A. pullulans* [79], potassium aluminum sulfate [78] and streptomycin sulfate [44] should enable the high cultivar diversity present in Swiss apple orchards to be preserved. Experts stated that the lower efficacy observed for some products (some copper compounds and aluminum sulfate [6,75]) does not affect overall efficacy, since these products are only used in combination with other phytosanitary products and under particular weather conditions. In contrast, experts estimated that the use of FB-resistant/tolerant apple cultivars (classically bred and molecularly engineered) may result in the reduction of cultivar diversity, since they might replace highly FB-susceptible cultivars. However, it was difficult for the experts to estimate the impact of FB-resistant cultivars on cultivar diversity since, from an economic point of view, “Golden Delicious”, “Gala” and “Braeburn” are still the most convenient apples to produce. Second, these cultivars and the related agricultural practices are relatively well known (production, year to year variation, plant structure, marketing, storage *etc.*). Moreover, new pesticides are cheaper than new cultivars, and are therefore preferred by the growers. This means that as long as some effective pesticides (e.g., streptomycin) are available, producers would rather stay with the current popular cultivars promoted by the apple marketers. Trends in Switzerland show that the production of “Gala” is increasing, while that of “Golden Delicious” is decreasing.

The diversity of agricultural practices (integrated production, organic production, standard trees, dwarf trees) may be guaranteed by the use of *A. pullulans* and classically bred or genetically engineered resistant apple cultivars. However, the use of copper compounds, potassium aluminum sulfate and streptomycin sulfate may result in only partial protection of existing agricultural practices [45]. While streptomycin sulfate is not permitted in Swiss organic farming [59] (allowed in organic farming in the US until 2014), potassium aluminum sulfate is likely to become available in the near future. However, organic apple production aims to adopt tolerant/resistant cultivars. Experts further stated that the impact of FB-resistant cultivars on standard apple tree production is still an open question. Standard apple tree production is considered hard to manage: working practices are difficult and few pesticides are applied (need for special application tools). These difficulties have resulted in a decrease in standard apple tree production in Switzerland over the last 100 years. This has been reinforced by low prices for juice and cider raw material. If an old standard tree is eradicated and the farmer wants to plant another standard tree, choosing an FB-resistant or tolerant cultivar is recommended. Thus, to prevent the disappearance of standard tree apple production, it is recommended that nurseries produce and sell FB-tolerant standard apple trees such as “Schneiderapfel” or “Alant”, in order to avoid the replant of highly FB-susceptible cultivars such as “Berner Rosenapfel”. However, in the Canton of Aargau for example, the cantonal authorities also promote the planting of broadleaf trees (e.g., *Tilia*) to replace old standard fruit trees. This strategy maintains a similar level of biodiversity, while further decreasing standard tree apple production. Another strategy (e.g., used in Aargau and Thurgau) includes planting and preserving standard trees in some parts of the canton while favoring the growth of dwarf tree orchards in other parts. The Canton of Aargau, for example, is divided into two zones: about 25% of the land (in the southern part of the canton) is contaminated by FB. Here, symptoms are monitored, but infected plants are not systematically eradicated and a pruning strategy aimed at lowering pathogen pressure is performed. However, in the other 75% of the surface area (the northern part of the canton), the systematic monitoring of symptoms is followed by a consequent eradication of infected plants, in order to keep this part FB-free. Since every infected tree has to be removed, standard apple tree production is not really practicable anymore. The monitoring of the whole cantonal land is very costly but important. It is essential to find as many infected plants as possible, in order to decide on the right control strategy (eradication or pruning). It is also of the utmost importance to eradicate wild and ornamental host plants, without which no FB control strategy will work. Unfortunately, complete eradication of these host plants is not feasible: experts stated that about 4 million m^2^ *Cotoneaster* are present in the Canton of Aargau, and the costs for complete eradication action would be too high.

### 3.2. Summary of Results

The results of this study, obtained through a literature search and interviews with experts, are summarized in Table 3a. Results of the questionnaire are summarized in Table 3b. Due to the complexity of the questionnaire, the wide range of topics and missing data, experts felt comfortable answering only a few questions, resulting in just 2–5 assessments per individual question. Despite the sparse answers provided by the experts, the results of literature search, interviews with experts and the questionnaires were comparable (Table 3a,b). The following differences have been identified: (1) experts think that the efficacy of *A. pullulans* is lower than the literature tells us; (2) information based on literature searches suggest that copper and aluminum compounds will have a greater impact on the soil and water environments than the experts have forecast. This is probably due to the fact that in Switzerland apple orchards are on basic soils, which decreases the bioavailability and increases accumulation of these metals; (3) experts think that the application of streptomycin may have an important impact at the biodiversity level (especially on blue-green algae and the selection of antibiotic-resistant bacteria), while field studies reviewed here suggest the restricted use of this antibiotic in Swiss orchards (at the moment one application per year maximum) should not pose any particular risk; and (4) experts forecast potential risks for the diversity of agricultural practices by using genetically engineered apple plants. This is probably due to the fact that it is not yet clear in which agricultural settings (organic, IP, standard trees, dwarf trees) genetically engineered FB-resistant apple plants would be used.

Table 3Overview of the assessment of fire blight (FB) control methods: classification of identified uncertainties and problems.

**Table:** 

(a) Summary Based on Literature Search and Interviews with Experts										
Protection Goals	Fire Blight Control Measures									
		Biological Control	Chemical Control				Conventional Breeding ^a^		GMO ^b^	
	Commercial Name Composition	Blossom Protect *A. pullulans*	Copper	Myco-sin Aluminum Sulfate	LMA Potassium Aluminum Sulfate	Strepto, Firewall, Streptomycin Sulfate	1 gene	2 gene	1 gene	2 genes
							“Ladina” *FB_F7* QTL	*FB_F7* QTL + ??	“Gala” + *FB_MR5* ^c^	“Gala” + *FB_MR +* ??
**FB-free agricutural crop and environment**										
Feasibility		E ^d^	E	E	E	E	E	possible	E	possible
Efficacy of method		73–78%	34–88%	51–65%	73%	76–89%	60–75%	? ^e^	93–100%	?
Durability		high	high	high	high	resistant strains?	virulent strains?	virulent strains?	virulent strains?	virulent strains?
**Protection of consumer and workers **		AT ^f^	AT	AT	AT	AT + resistance? ^g^	Allergy?		?	?
							no tests required			
**Protection of environment**										
Impact on exposed animals		low	low	low	low	low	low	low	low	low
Impact on biodiversity		low	medium	medium	medium	low	low	low	low	low
Impact on soil and water		low	medium	medium	medium	medium	low	low	low	low
**Economic interest (acceptance)**										
Quality accepted and desired by consumer		medium	medium	high	high	medium	high	high	low	low
Way of production acceptable for consumer		high	high	high	high	medium	high	high	low	low
**Maitain cultivar diversity and diversity of cultivation practices**										
Impact of method on cv diversity		low	medium	medium	low	low	high	high	high	high
Impact of method on cultivation practices		low	medium	medium	medium	medium	low	low	low	low

Green: no or negligible problems identified; Yellow: minor uncertainties and/or minor problems identified; Orange: Uncertainties and/or problems identified that urgently need to be addressed. **^a^**: Conventional breeding: apple cultivars carrying a one or two gene-based FB resistance obtained by conventional breeding; **^b^**: GMO: Genetically Modified Organism carrying a one or two gene-based FB resistance; **^c^**: “Gala” + *FB_MR5*: estimations based on transgenic lines tested under greenhouse conditions with two *E. amylovora* strains; **^d^**: E: Product already exists; **^e^**: ?: Unknown; **^f^**: AT: Safety of product tested for registration. If used according to instructions no problems for workers and consumers; **^g^**: Acquisition of antibiotic (multi) resistant bacteria in operators not tested.

**Table:** 

(b) Summary of Questionnaires Answered by Experts										
Protection Goals	Fire Blight Control Measures									
		Biological Control	Chemical Control				Conventional Breeding ^a^		GMO ^b^	
	Commercial Name Composition	Blossom Protect *A. pullulans*	Copper	Myco-sin Aluminum Sulfate	LMA Potassium Aluminum Sulfate	Strepto, Firewall, Streptomycin Sulfate	1 gene	2 gene	1 gene	2 genes
							“Ladina” *FB_F7* QTL	*FB_F7* QTL + ??	“Gala” + *FB_MR5* ^c^	“Gala” + *FB_MR5 +* ??
**FB-free agricutural crop and environment**										
Feasibility		E ^d^	E	E	E	E	E	possible	E	possible
Efficacy of method		medium	medium	medium	medium	high	high	? ^e^	high	?
Durability		high	high	high	high	low	medium-low	medium	medium-low	medium
**Protection of workers **		low	low	low	low	?	low	low	low	low
**Protection of consumer**		low	low	low	low	low	low	low	low	low
**Protection of environment**										
Impact on exposed animals		low	low	low	low	low	low	low	low	low
Impact on biodiversity		low	medium	medium	medium	high	low	low	low	low
Impact on soil and water		low	low	low	low	medium	low	low	low	low
**Economic interest (acceptance)**		high	high	high	high	medium	high	high	low	low
Quality accepted and desired by consumer										
Way of production acceptable for consumer										
**Maitain cultivar diversity and diversity of cultivation practices**										
Impact of method on cv diversity		medium	medium	medium	low	low	high	high	high	high
Impact of method on cultivation practices		?	?	?	?	high	low	low	medium	medium

Green: no or negligible problems identified (severity 1 and probability of damages 1–4 or severity 2 and probability of damages 1); Yellow: minor uncertainties and/or minor problems identified (severity 2 and probability of damages 2–4 or severity 3 and probability of damages 1); Orange: Uncertainties and/or problems identified (severity 3 and probability of damages 2–4 or severity 4 and probability of damages 1–4). **^a^**: Conventional breeding: apple cultivars carrying a one or two gene-based FB resistance obtained by conventional breeding; **^b^**: GMO: Genetically Modified Organism carrying a one or two gene-based FB resistance; **^c^**: “Gala” + *FB_MR5:* “Gala” + *FB_MR5*: estimations based on transgenic lines tested under greenhouse conditions with two *E. amylovora* strains; **^d^**: E: Product already exists; **^e^**: ?: Unknown.

## 4. Conclusions

In summary, our study based on literature searches and interviews with experts concludes that the FB control measures currently applied in Switzerland are largely safe for consumers, workers and the environment. Yet, we come to the conclusion that there are still several important gaps regarding human/animal health and environment, depending on the different FB control measures used (or applied). These gaps cause considerable uncertainty in the assessment, and therefore need to be addressed in the near future. In the long-term, most of the FB control products used at present, although successfully applied over the last few years, are probably not efficient enough to ensure safe apple production, especially in organic production if we continue to plant susceptible cultivars and if in future years infection pressure reaches again levels observed in 2007. Moreover, the more effective pesticides, streptomycin and potassium aluminum sulfate, are not allowed in organic apple production. Therefore, there is an urgent need for resistant cultivars and for new products that are more efficient and durable, and are also compatible with organic apple production.
